# Assessment of alterations in regional homogeneity and amplitude of low-frequency fluctuations in children with dyslexia

**DOI:** 10.55730/1300-0144.6072

**Published:** 2025-07-26

**Authors:** Esra DEMİRCİ, Şerife GENGEÇ BENLİ, Semra İÇER, Zeynep AK, Ebru AKER, Zehra Filiz KARAMAN

**Affiliations:** 1 Department of Child and Adolescent Psychiatry, Faculty of Medicine, Erciyes University, Kayseri, Turkiye; 2 Department of Biomedical Engineering, Engineering Faculty, Erciyes University, Kayseri, Turkiye; 3 Department of Biomedical Engineering, Graduate School of Natural and Applied Sciences, Erciyes University, Kayseri, Turkiye; 4 Department of Pediatric Radiology, Faculty of Medicine, Erciyes University, Kayseri, Turkiye

**Keywords:** Dyslexia, special education, rs-fMRI, exploratory analyses

## Abstract

**Background/aim:**

Exploratory methods in neuroimaging are gaining increasing attention for revealing functional connectivity structures across the entire brain. This study aims to contribute to the understanding of the functional mechanisms underlying dyslexia, along with the effects of specialized education for dyslexia treatment, by utilizing measures such as regional homogeneity (ReHo), amplitude of low-frequency fluctuations (ALFF), and fractional ALFF (fALFF).

**Materials and methods:**

Resting-state functional magnetic resonance imaging data were collected from three groups of Turkish-speaking children aged 7–12: diagnosed with dyslexia for the first time (Dys, n = 19), children with dyslexia who received specialized education (EDys, n = 20), and healthy controls (HC, n = 27). In the study, brain activation in individuals with dyslexia was examined through resting-state analyses employing the ReHo, ALFF, and fALFF methods.

**Results:**

Significant reductions in ReHo and ALFF values were observed in brain regions associated with phonological processing and visuomotor integration in children with dyslexia. These findings indicate altered neural synchronization related to cognitive deficits in reading and language processing. Compared to HC, children with Dys showed significantly reduced ReHo and ALFF values in the left precuneus and middle frontal gyrus, while those EDys exhibited compensatory increases in calcarine and lingual gyri.

**Conclusion:**

This study provides valuable insights into the resting-state neural connectivity of individuals with dyslexia, highlighting the impact of specialized educational programs in treating dyslexia. Our findings contribute to understanding the altered connectivity foundations of dyslexia compared to healthy children and support the development of educational interventions within this framework.

## 1. Introduction

Dyslexia is a neurodevelopmental disorder characterized by difficulties in reading, writing, and language processing abilities [1,2]. Dyslexia affects approximately 5%–15% of children worldwide and around 4% of adults, significantly impacting individuals’ academic, social, and professional lives [3]. In recent years, neuroimaging techniques have played a crucial role in understanding the neurobiological foundations of dyslexia [4–6]. Investigating brain activities in the resting state through resting-state functional magnetic resonance imaging (rs-fMRI) is critical for uncovering how dyslexia affects functional connectivity in the brain [7,8].

fMRI studies on individuals with dyslexia reveal decreased activation in certain brain regions associated with language and reading skills [9–11]. Specifically, reduced activity has been observed in the left temporo-parietal region, which is essential for phonological processing and reading, and in the left inferior frontal gyrus (Broca’s area), which supports speech production and syntax [12–14]. Similarly, reduced activation has been found in the dorsal and ventral language pathways during phonological processing and comprehension tasks [15,16]. These findings highlight the need to understand the neurobiological mechanisms shaping dyslexia-related functional connectivity differences in the brain that contribute to reading difficulties.

Within rs-fMRI analysis methods, Regional Homogeneity (ReHo), Amplitude of Low-Frequency Fluctuations (ALFF), and fractional ALFF (fALFF) analyses stand out for their ability to evaluate synchronized activity across brain regions. ReHo analysis measures the homogeneity of local functional connectivity in the brain, while ALFF and fALFF analyses assess the strength of resting-state brain activities by evaluating the amplitude of low-frequency fluctuations [17–19]. This study aims to use these methods to determine whether there are differences in resting-state brain activities of individuals with dyslexia and to examine how these differences might influence the cognitive processes associated with dyslexia. ReHo, ALFF, and fALFF analyses in resting-state fMRI studies reveal that individuals with dyslexia exhibit reduced local connectivity and that low-frequency activity in certain regions is associated with dyslexia-specific cognitive impairments [20,21].

In the resting-state and task-based fMRI study conducted by Yang et al., the visual cortex (V1) region was found to have significant functional connectivity in both dyslexic and control groups during rest. In the control group, strong negative correlations were observed between the functional connections of the left superior frontal gyrus (SFG) and the middle frontal gyrus (MFG), whereas these correlations were found to be weaker in the dyslexic group [22]. In the resting-state fMRI study by Hashimoto et al., where the left inferior frontal gyrus (IFG), left inferior parietal lobule, left and right fusiform gyrus, and right inferior occipital gyrus were selected as seed regions, no significant differences in resting-state functional connectivity were observed between the dyslexia and control groups. A seed-based analysis was conducted to examine the correlation of time series from the relevant ROIs with Hiragana fluency and Kanji accuracy [7]. In the resting-state and task-based fMRI study conducted by Schurz et al. with dyslexic and typical readers, it was found that the functional connectivity between the left posterior temporal regions and the IFG was weakened in dyslexic readers. Specifically, a reduced connection between the left fusiform gyrus and ITG and the IFG pars triangularis and pars opercularis was observed in dyslexic readers. Additionally, stronger connections were detected between reading-related brain regions and the default mode network (precuneus) in dyslexic readers [23]. In the resting-state fMRI study by Zhou et al., the functional connectivity (FC) networks in the dorsal and ventral visual regions were identified in both control and dyslexia groups, with a focus on two key nodes: the left intraparietal sulcus (IPS) and the visual word form area (VWFA). The role of these regions in reading was assessed, and it was found that the left IPS and VWFA exhibited a similar FC pattern with bilateral ITG, IPS, and left MFG, whereas in dyslexic children, these connections were weakened within the network [24].

In a study conducted by Li et al., it was noted that in children with dyslexia, there were alterations in ReHo values in certain brain regions, which indicated impaired local brain activity synchronization. These alterations may contribute to the difficulties they face in reading and other related cognitive tasks. Specifically, it was found that children with dyslexia had lower ReHo values in areas related to language processing and visual-motor integration, which could potentially contribute to the assessment of reading fluency and accuracy [20]. The findings of a study by Song et al. suggest that ReHo values, along with other metrics such as fALFF and degree centrality, help in understanding the maturation of brain regions critical for phonological processing and rapid naming. Higher ReHo values in specific areas, such as the PCC and IFG, are associated with developing language skills, highlighting the importance of these regions in language development [21].

Research on dyslexia has shown that special education and individualized learning approaches play a crucial role in the language and cognitive development of these individuals [25]. Special education support helps individuals with dyslexia develop their skills by focusing on their weaknesses while also highlighting their strengths, thereby fostering self-confidence. High-quality special education programs include systematic and structured techniques aimed at improving reading and writing competencies, assisting individuals in adapting to their learning experiences [26,27]. The implementation of such education at an early age plays a critical role in reducing the effects of dyslexia by enabling individuals to achieve greater success in their educational and social relationships [28]. In the review conducted by Martins et al. (2025), it was reported that the majority of rs-fMRI studies in the field of dyslexia rely on hypothesis-driven analytical methods, such as seed-based functional connectivity. In contrast, data-driven approaches like ReHo and ALFF have been employed in only a limited number of studies. Furthermore, clinical intervention variables such as specialized education have rarely been considered in neuroimaging analyses [29].

In this context, our study constitutes the first in the literature to incorporate both ReHo/ALFF analyses and participants who have received specialized educational interventions. The unique contributions of this study are outlined below:

- This study aims to apply a variety of exploratory connectivity analyses to reveal the intrinsic structure of the brain at rest, independent of task demands.
- According to our research, this is the first study to employ exploratory fMRI techniques such as ReHo, ALFF, and fALFF in examining children with dyslexia who received specialized education.
- In this context, the study is the first to include three groups: children diagnosed with dyslexia for the first time, children with dyslexia who received specialized education, and typically developing controls, in order to identify differences in brain activation.
- The comparisons of functional connectivity between groups elucidate how dyslexia alters brain function and how educational intervention modulates these alterations.
- The findings obtained from this study not only contribute to a better understanding of dyslexia but also provide valuable insights for the development of more effective treatment and intervention strategies.

## 2. Materials and methods

### 2.1. Participants

This study analyzed rs-fMRI data from 66 native Turkish-speaking children divided into three groups: 19 diagnosed with dyslexia for the first time, (Dys), 20 who had received special education for dyslexia (EDys), and 27 healthy controls (HC). Participants were aged 7–12, right-handed, and had IQ scores above 80.

Among the participants evaluated using the Kiddie Schedule for Affective Disorders and Schizophrenia–Present and Lifetime Version–DSM-5–Turkish Version (K-SADS-PL-DSM-5-T) [30] and through clinical interviews, only children without any known chronic illness, without comorbid psychiatric disorders, and meeting diagnostic criteria solely for dyslexia among specific learning disorders were included in the study.

To assess the various cognitive abilities of all participants, the Wechsler Intelligence Scale for Children–Fourth Edition (WISC-IV) was administered individually. Children in the Dys and EDys group were diagnosed with dyslexia for the first time, based on DSM-V criteria and the Turkish national Specific Learning Disability (SLD) diagnostic battery.

In addition, the EDys group consisted of children who had participated in individualized education programs for a duration of one to two years, in accordance with the criteria set by the Turkish Ministry of National Education [31] and who had obtained a Special Needs Child Report (ÇÖZGER).

The HC group was composed of children who visited outpatient clinics for counseling purposes and were confirmed to have no psychiatric disorders. Clinical characteristics and sociodemographic information of the participants, along with statistical significance levels, are presented in Table 1.

### 2.2. Data acquisition

Functional and structural MR images of each participant were acquired using a Siemens Magnetom Aera 1.5 Tesla MRI scanner with a 20-channel head coil [32,33]. Structural MR images were acquired using a T1-weighted MPRAGE (magnetization-prepared rapid gradient-echo) sequence. The scanning parameters were set as follows: sagittal orientation, echo time (TE) = 2.670 ms, repetition time (TR) = 1900 ms, 256 × 256 matrix, isotropic resolution = 1.3 mm, flip angle = 15°, and total scan time = 4 min 18 s for 192 slices. These structural MR images were used during the preprocessing of functional images to align the participants’ brain anatomy [34]. Functional MRI data were acquired using a T2*-weighted echo-planar imaging sequence with the following parameters: TR = 2800 ms, TE = 53 ms, flip angle = 90°, field of view (FOV) = 192 mm, 25 slices covering the entire brain, slice thickness = 5 mm, and in-plane resolution = 2 × 2 mm. The resting-state functional data used in this study were acquired for 6 min and 14 s, consisting of 130 volumes [33]. During the resting-state fMRI scan, participants were instructed to keep their eyes open and remain still without thinking about anything.

**Figure 1:**
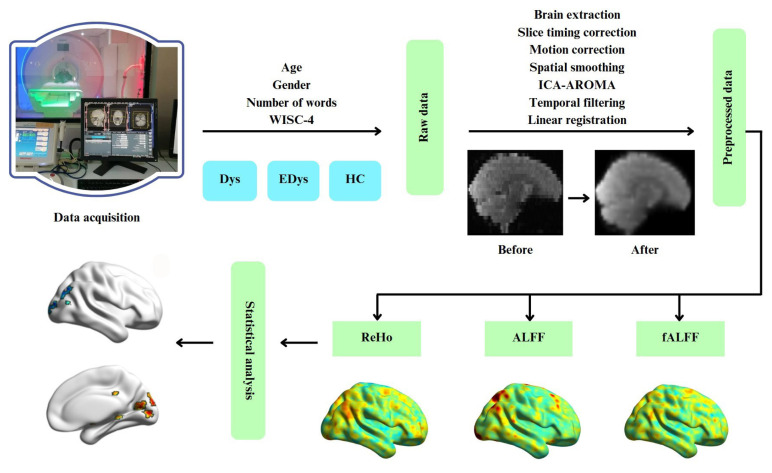
Flowchart of the study process. Abbreviations: *Dys: Children diagnosed with dyslexia for the first-time, *EDys: Children with dyslexia who received special education, *HC: Healthy controls, *ReHo: Regional homogeneity, *ALFF: Amplitude of low-frequency brain oscillations, *fALFF: fractional ALFF.

Throughout the study, structural and functional MRI data were acquired from male and female children aged 7–12. The process was subjected to preprocessing in order to test the suitability of the data and make it appropriate for analysis. Resting-state brain connectivity differences were examined by applying ReHo, ALFF, and fALFF analyses to the preprocessed data. Following the analyses, brain maps for each participant were evaluated using statistical analysis to investigate the differences between groups. The steps followed from data acquisition to analysis are summarized in a flowchart of the study process in Figure 1.

### 2.3. Preprocessing

The preprocessing steps of the functional MR images were performed using the FMRIB Software Library (FSL 6.0.4) (https://fsl.fmrib.ox.ac.uk/fsl/fslwiki). The preprocessing process included the following steps: brain extraction, slice timing correction, motion correction, spatial smoothing, ICA AROMA, temporal filtering, and linear registration [33]. First the brain extraction tool (BET) from FSL was used to separate non-brain structures from the anatomical and functional images [35]. Subsequently, slice timing correction was applied to align all slices as if they were acquired simultaneously. Motion correction was performed by using the middle slice as a reference and applying 6 degrees of freedom (3 translations and 3 rotations) using the FLIRT method [36]. Children with estimated maximum head motion greater than 2 mm/° or average motion greater than 0.5 mm/° were excluded from the study to reduce unwanted motion-related effects [33]. Spatial smoothing was performed by averaging the values of neighboring voxels to reduce high-frequency fluctuations. After motion artifacts were detected and removed using the ICA-AROMA tool, temporal filtering was applied to clean the high-frequency signals [37]. Functional data were linearly registered to high-resolution anatomical images using FLIRT [36]. Structural images were registered to the 2-mm MNI standard space template using FNIRT with 12 degrees of freedom and then combined with the low-resolution fMRI images [33].

### 2.4. Regional homogeneity (ReHo) analysis

Regional homogeneity (ReHo) analysis is an fMRI analysis method used to measure the local homogeneity of neural activity in specific brain regions [38]. This method provides information about the consistency of brain functions in local regions and is used to investigate the effects of conditions such as psychiatric disorders on the brain [21]. ReHo analysis was performed using the Data Processing Assistant for Resting-State fMRI (DPARSF) (http://rfmri.org/DPARSF) [39] and the Kendall’s coefficient of concordance (KCC) was calculated. The ReHo value for each voxel was determined by calculating the KCC of the time series of its 27 nearest neighboring voxels [40]. Subsequently, the ReHo value for each voxel was standardized by dividing it by the global mean ReHo value of the individuals. Then, the fMRI data were smoothed using a 5 mm FWHM Gaussian Kernel.

### 2.5. ALFF/fALFF analysis

Studies in the literature suggest that low-frequency fluctuations observed in resting-state fMRI BOLD signals contain physiologically meaningful information and are closely related to spontaneous neuronal activities [41,42]. Zang et al. defined the mean amplitude of low-frequency fluctuations (ALFF) as an indicator of spontaneous neuronal activity. ALFF is typically defined as the square root of the average power spectral density of the BOLD signal within the 0.01–0.08 Hz frequency range. After obtaining individual ALFF maps, a standardization process is applied, where each ALFF value is divided by the average ALFF value within a specific mask (the whole brain or a user-defined mask), similar to the procedure used in ReHo analysis [43].

Although ALFF reflects the regional intensity of spontaneous brain activity, it may be affected by physiological noise in some brain regions. Fractional ALFF (fALFF), an enhanced version of ALFF calculated as the ratio of the power spectrum of low-frequency signals to the entire frequency range, is more effective in suppressing high-frequency noise [44].

### 2.6. Statistical analysis

Statistical analyses of the functional data used for connectivity analysis were conducted using DPARSF 5.4 (http://rfmri.org/dpabi) and SPM12 (http://www.fil.ion.ucl.ac.uk/spm). To examine group differences in the data obtained from the ReHo analysis, the Analysis of Covariance (ANCOVA) method was employed. ANCOVA is a statistical technique used to evaluate the effects of independent variables and one or more covariates on a dependent variable. ReHo, ALFF, and fALFF values were defined as dependent variables, while the groups (Dys, EDys, and HC) were included in the model as independent variables. Differences in functional connectivity values among the groups were evaluated, along with the potential influence of covariates age and gender. When ANCOVA revealed significant group differences, post-hoc tests were conducted to determine which specific groups differed from each other [45]. While the group mean values of ReHo and ALFF analyses show a similar pattern, differences are observed in the fALFF analysis [46]. Figures 2a–2c show the distributions of mean ReHo, ALFF and fALFF values for each group.

## 3. Results

This study investigates the differences in functional connectivity between Dys, EDys, and HC groups using exploratory methods based on spontaneous brain activity, namely ReHo, ALFF, and fALFF. The findings for each method are presented in the following subsections.

### 3.1. Results of ReHo analysis

**Figure 3:**
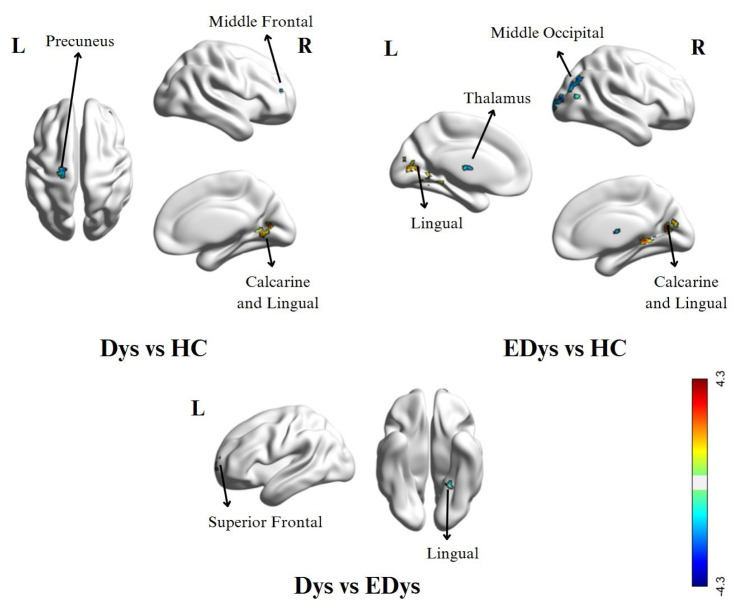
Brain activity differences between groups in ReHo analysis. Abbreviations: *Red activation indicates the first group > second group, while blue activation indicates the second group > first group under the visuals. *Dys: Children diagnosed with dyslexia for the first-time, *EDys: Children with dyslexia who received special education, *HC: Healthy controls.

In the Dys group, higher activity was observed in the right calcarine and right lingual regions compared to the HC group. In contrast, the HC group exhibited greater activity in the left precuneus, right middle frontal, and left precentral regions compared to the Dys group. In the EDys group, increased activation was observed in the left lingual and right lingual regions, as well as the right calcarine region, compared to the HC group. Conversely, the HC group showed higher activity in the right middle occipital, left middle temporal, left thalamus, and right middle temporal regions compared to the EDys group. In the Dys group, greater activation was observed in the left upper temporal region compared to the EDys group. Finally, the EDys group showed an increase in activation in the left lingual and left upper frontal regions compared to the Dys group. The results of the ReHo analysis, detailing the brain activity differences between the Dys, EDys, and HC groups, are presented in Table 2, with corresponding visual representations shown from different angles in Figure 3.

### 3.2. Results of ALFF analysis

**Figure 4:**
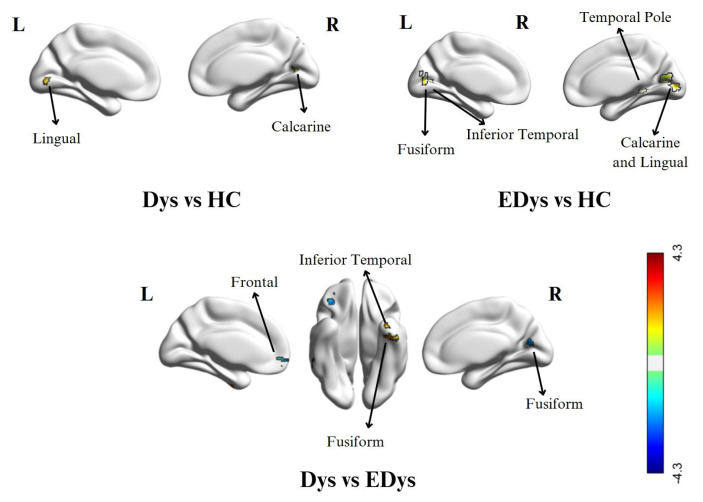
Brain activity differences between groups in ALFF analysis. The explanations in Figure 3 are valid.

In the Dys group, compared to the HC group, significant increases in ALFF were observed in the left lingual gyrus, right calcarine, and vermis 7, In contrast, the HC group showed higher activity in the right cuneus. regions compared to Dys. In the EDys group, significant activity increases were found in the left cerebellum, right calcarine, and right lingual gyrus regions compared to HC. Compared to the HC, the EDys group showed more activity increases in the left fusiform gyrus, right middle temporal pole, and left inferior temporal regions. Furthermore, in the Dys group, activation increases were observed in the left and right fusiform gyrus, right cerebellum 6, and inferior temporal regions compared to the EDys group. In contrast, the EDys group exhibited more activity increases in the right and left frontal, orbitofrontal, and right calcarine regions compared to the Dys group. In the ALFF analysis, brain activity differences between the Dys, EDys, and HC groups are presented in Table 3 and visually expressed in Figure 4.

### 3.3. Results of fALFF analysis

**Figure 5:**
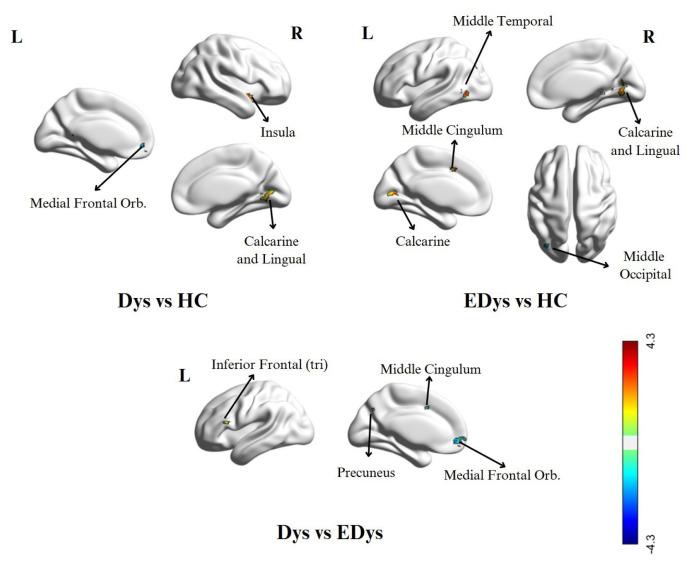
Brain activity differences between groups in fALFF analysis. The explanations in Figure 3 are valid.

In the Dys group, higher activity was observed in the right insula, right calcarine, and right lingual regions compared to HC. Conversely, the HC group exhibited more activity increases in the left orbitofrontal, left precuneus, and right postcentral regions. In the EDys group, significant activity increases were observed in the left middle temporal, right calcarine, right lingual, and left calcarine regions compared to HC. Compared to the EDys group, the HC group showed activity increases in the right middle temporal and left middle occipital regions. In the Dys group, more activity was observed in the left inferior frontal region compared to the EDys group, while the EDys group showed activity increases in the left cerebellum, left orbitofrontal, left precuneus, and left middle cingulum regions. In the fALFF analysis, brain activity differences between the Dys, EDys, and HC groups are presented in Table 4 and visually expressed in Figure 5.

## 4. Discussion and conclusions

In recent years, the growing use of fMRI to study neuropsychiatric disorders has led to increased research focused on understanding the functional basis of dyslexia and reading difficulties. In this context, the primary objective of this study is to investigate the neuronal activity alterations in the brain caused by dyslexia, one of the most common childhood disorders today, through exploratory analyses such as ReHo, ALFF, and fALFF. Additionally, another key aspect of the study is to contribute to understanding the alterations in brain function caused by the education provided in dyslexia.

In the ReHo analysis, increased regional homogeneity was observed in the right calcarine and lingual gyri in the Dys group. In contrast, the HC group showed stronger activity in the precuneus and middle frontal regions. In the EDys group, activity increases in the calcarine and lingual regions suggest that education may enhance connectivity in these areas.

In the ALFF analysis, the increased activation in the lingual gyrus and calcarine regions in the Dys group, and in the cerebellum and temporal regions in the EDys group, reflect the changing patterns of brain activity at different stages of dyslexia. The increase observed in the frontal regions in the EDys group may indicate functional improvements in connectivity due to education.

Similarly, in the fALFF analysis, higher activation was observed in the insula and lingual regions in the Dys group, while significant increases were detected in the orbitofrontal and precuneus regions in the HC group. The increase in activation in the orbitofrontal and temporal regions in the EDys group suggests that special education for dyslexia may support functional adaptations in these areas.

Overall, the findings indicate that brain activity in Dys and EDys groups may change through the educational process, and that these connections can be strengthened through intervention. However, studies on dyslexia during resting state in the literature are quite limited compared to task-based studies, and there are almost no studies involving ReHo, ALFF, and fALFF analyses in this context. This gap provides clear evidence that brain activity patterns related to dyslexia need to be further explored under resting conditions.

The findings of limited studies on resting-state fMRI in dyslexia that we encountered during our literature review are evaluated in conjunction with the results of our study. In the study by Kuhl et al., brain functional connectivity was examined using ReHo analysis to assess the reading and writing abilities of a dyslexic group before and after intervention. The study focused on the following regions of interest (ROIs): left primary visual cortex, left middle temporal area, left fusiform gyrus, left primary auditory cortex, left planum temporale, left ventral premotor cortex, and left inferior frontal gyrus. The results showed that in dyslexic children, the functional connectivity between the left primary auditory cortex and the left planum temporale was found to be lower compared to the control group [47]. In another study, during resting-state fMRI, the dyslexic group showed stronger functional connectivity in the left occipitotemporal gyrus compared to the control group [48]. In this study, greater activation in the fALFF was observed in the left middle occipital region in the HC group compared to the EDys group. In another study, a stronger connection was found between the left middle temporal gyrus and the left calcarine sulcus in dyslexic readers compared to healthy readers [23]. Additionally, in dyslexic readers, a stronger connection was found in the precuneus region between reading-related areas and default mode network areas compared to healthy readers [23].

Across all three analyses in this study, greater activation was observed in the right calcarine region in the dyslexic groups (Dys and EDys) compared to HC group. Additionally, in the ALFF analysis, greater activation was found in the same region for EDys>Dys, while in the fALFF analysis, EDys>HC showed greater activation in the left calcarine. This suggests that the calcarine region has the potential to form a strong functional area of interest for dyslexia.

In a resting-state study using Granger Causality Analysis, a significant decrease in effective connectivity was observed in the dyslexia group compared to the healthy control group. Specifically, reductions were seen in the connections between the left precentral gyrus and right precuneus, the left anterior cingulate gyrus and paracingulate gyrus, the left calcarine and right angular gyrus, and the left middle frontal gyrus and left calcarine. Additionally, an increase in connectivity was detected between the right cuneus and left inferior frontal gyrus triangularis in the dyslexia group [49]. In our study, the left precuneus region showed increased activation in the fALFF analysis in the HC>Dys and EDys>Dys comparisons, while the ReHo analysis revealed increased activation in HC>Dys. In the left precentral gyrus region, increased activation was observed in the EDys>HC comparison in the ReHo analysis.

Greeley et al. observed an increased functional connectivity between the sensorimotor network and the left angular gyrus, left lateral occipital cortex, and right inferior frontal gyrus in the dyslexia group. Additionally, a decreased functional connectivity between the cerebellar network and the precentral gyrus was detected. Seed-based analysis results revealed that in the dyslexia group, there was an increase in functional connectivity between the cerebellum, including Crus 1, lobule 6, and lobule 8, and brain regions associated with the default mode network and motor system [50].

In this study, the ALFF analysis revealed increased activation in the left cerebellum (lobule 8) and vermis 7 regions in the comparison of EDys>HC, and increased activation in the right cerebellum (lobule 6) in the comparison of Dys>EDys. Despite the limited studies on dyslexia, functional connectivity alterations in the cerebellum associated with dyslexia have been scarcely investigated in the literature. Our findings provide potential evidence that the cerebellum should be considered as one of the regions to be explored in dyslexia research.

In another study by Horowitz-Kraus et al., resting-state fMRI was used to evaluate functional connectivity in children with reading difficulties and a control group, prior to and following a reading intervention. The study found an increase in functional connectivity within the fronto-parietal network both before and after the reading intervention in both dyslexic and typical readers, with the reading difficulties group showing greater improvement. Statistical analysis revealed that, compared to the control group, children with reading difficulties had significantly more functional connectivity in the cingulo-opercular network after the intervention. This was interpreted as suggesting the importance of cognitive control during reading in this population [51].

In addition to our literature review, another key finding from our study across all three analyses and group comparisons is the functional connectivity patterns in the lingual gyrus region. This area appears to be of significant importance for understanding and interpreting the alterations induced by dyslexia and the education provided to individuals with dyslexia.

Moreover, many of our findings are consistent with previously reported functional connectivity differences in dyslexia. In particular, alterations in activity within the precuneus, lingual gyrus, and temporal regions have been highlighted in prior resting-state fMRI studies [23,47]. However, increased activation observed in regions such as the right calcarine, insula, and cerebellum has been scarcely addressed in the existing literature, thereby distinguishing our study in this regard.

Resting-state fMRI is particularly valuable as it reveals the brain’s natural functioning and functional relationships without the need for a task-based paradigma. Exploring the spontaneous brain activity of dyslexia through methods such as ReHo, ALFF, and fALFF, without selecting specific ROIs or seed regions, can potentially provide valuable insights into the neuropsychiatric mechanisms underlying dyslexia. Resting-state fMRI studies in the field of dyslexia are quite limited, and to date, no study has employed ReHo, ALFF, and fALFF analyses to examine individuals who have received specialized education. Therefore, our study is particularly noteworthy as it is the first to investigate both dyslexia and the impact of specialized education using these three methods within a resting-state framework.

## 5. Limitations

Although the findings obtained in this study provide important contributions to the analysis of dyslexia using resting-state fMRI, certain methodological limitations should also be considered. First, in our study, the effects of special education on brain connectivity were not tracked within the same individuals; instead, the Dys and the EDys groups consisted of different participants. Therefore, we recommend that our findings be supported by long-term, longitudinal studies. Similarly, by including preeducation scores from various intelligence and psychometric scales for the special education group, comparisons could be made both in terms of connectivity and test scores before and after education. Statistically significant differences were found between groups in age, WISC-IV scores, and vocabulary scores, and these differences were controlled using ANOVA analysis. Children in the special education group received individualized programs over varying durations ranging from 12 to 24 months. Due to the personalized nature of these educational interventions, variations in duration and implementation may be considered an additional limitation. Moreover, sociodemographic data were collected using a form prepared by the researchers, rather than a standardized assessment tool. Despite these limitations, our study is the first to evaluate the effects of dyslexia and special education using ReHo, ALFF, and fALFF methods, thus making a significant contribution to literature.

## Figures and Tables

**Figure 2a:**
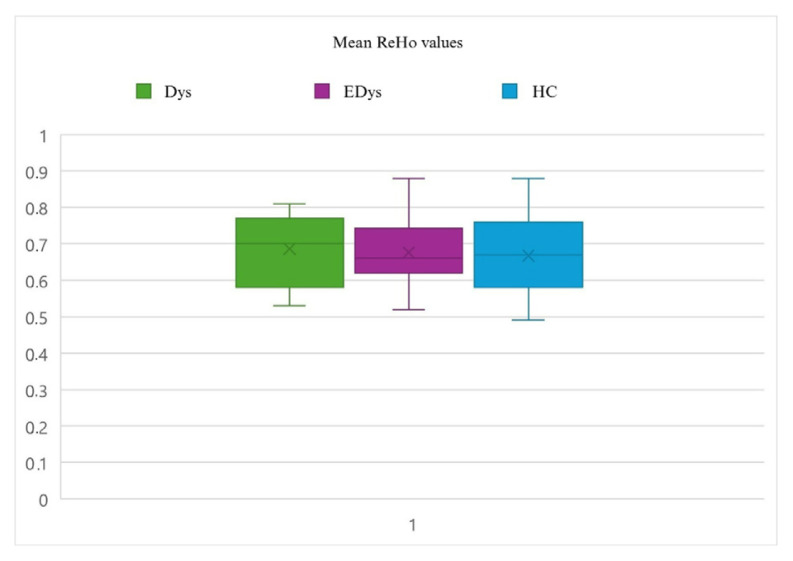
Distributions of mean ReHo values for each group. The explanations written in Figure 1 are valid.

**Figure 2b:**
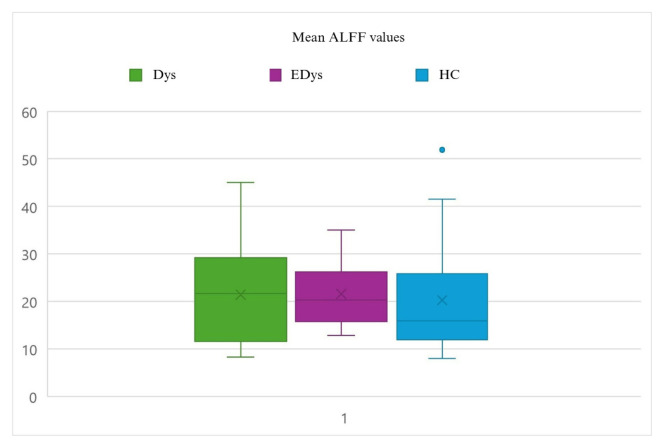
Distributions of mean ALFF values for each group. The explanations written in Figure 1 are valid.

**Figure 2c:**
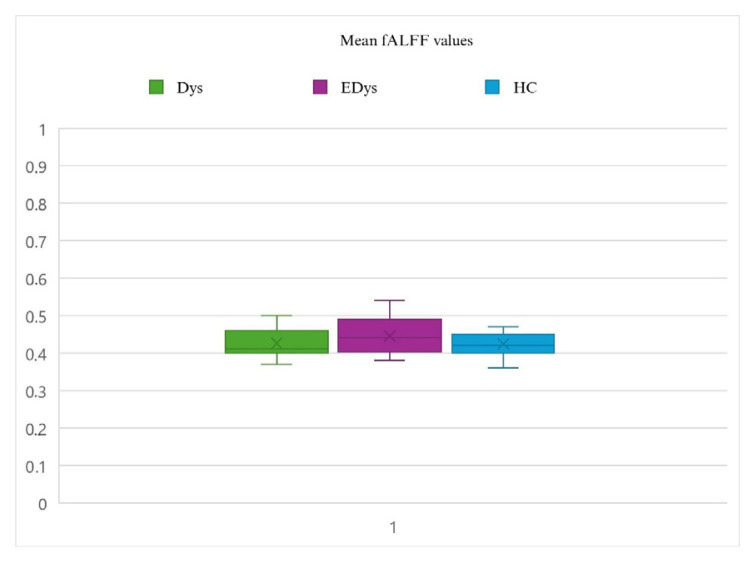
Distributions of mean fALFF values for each group. The explanations written in Figure 1 are valid.

**Table 1:** Clinical and sociodemographic information of the participants.

Clinical information	Dys	EDys	HC	p-value
**Sex (F/M)**	6/13	8/12	17/10	0.085
**Age (mean ± SD)**	8.63 ± 1.67*	9.58 ± 1.46	10.21 ± 1.51*	0.047
**Number of words (mean ± SD)**	25.58 ± 14.65*	38.26 ± 19.38*	89.21 ± 20.09*	<0.001
**WISC-IV (mean ± SD)**	86.47 ± 8.51*	91.32 ± 15.73	98.47 ± 8.96*	0.002
Mother’s age	40.38 ± 3.58	41.13 ± 4.39	39.78 ± 4.23	0.318
Father’s age	43.94 ± 4.34	44.18 ± 4.73	44.24 ± 5.07	0.372
Mother’s education level				
**Secondary school**	5 (26.3)	6 (30.0)	8 (29.6)	0.379
**High school**	10 (52.6)	9 (45.0)	11 (40.7)	
**University**	4 (21.0)	5 (25.0)	8 (29.6)	
Father’s education level				
**Secondary school**	2 (10.5)	3 (15.0)	3 (11.1)	0.118
**High school**	12 (63.1)	11 (55.0)	14 (51.8)	
**University**	5 (26.3)	6 (30.0)	10 (37.0)	
Parental marital status				
**Married**	18 (94.4)	20 (100.0)	26 (96.2)	0.405
**Divorced**	1(5.6)	0 (0.0)	1 (3.7)	
Number of siblings	1.79 ± 1.05	2.00 ± 0.85	1.74 ± 0.82	0.423

* The mean differences are significant at the 0.05 level. (Post Hoc Test: Tukey HSD)

Abbreviations: *Dys: Children diagnosed with dyslexia for the first time group, *EDys: Children with dyslexia who received special education, *HC: Healthy control group, WISC-IV: Wechsler Intelligence Scale for Children-Fourth Edition.

**Table 2:** ReHo analysis of brain activity differences between groups.

Groups	Brain regions (AAL Atlas)	Peak MNI coordinate			Voxel size	Peak intensity
		x	y	z		
**Dys > HC**	Calcarine R	12	−68	10	82	4.1467
	Lingual R	24	−60	2	85	4.2625
**HC > Dys**	Precuneus L	−2	−62	26	40	−3.402
	Frontal Mid. R	42	52	14	38	−3.4568
	Precentral L	−18	−20	70	32	−3.1097
**EDys > HC**	Lingual L	−24	−48	−4	34	3.3816
	Lingual L	−18	−66	−4	112	3.2005
	Lingual R	26	−46	−2	150	5.3916
	Calcarine R	12	−68	10	168	3.7518
**HC > EDys**	Occipital Mid. R	30	−92	14	45	−3.3508
	Thalamus L	−2	−10	8	32	−3.229
	Temporal Mid. L	−62	−58	22	37	−3.8579
	Temporal Mid. R	50	−70	16	63	−4.5178
	Occipital Mid. R	38	−78	28	66	−3.6673
**Dys > EDys**	Temporal Sup. L	−60	−62	16	40	3.3083
**EDys > Dys**	Lingual L	−24	−46	−4	30	−3.9117
	Frontal Sup. L	−20	66	0	63	−3.3941

* Dys: Children diagnosed with dyslexia for the first time group, *EDys: Children with dyslexia who received special education group, *HC: Healthy control group, *z > 2.3, *p < 0.05, *R: Right, *L: Left, *Mid: Middle, Sup: Superior, Cluster size > 30 voxels.

**Table 3:** ALFF analysis of brain activity differences between groups.

Groups	Brain regions (AAL Atlas)	Peak MNI coordinates			Voxel size	Peak intensity
		x	y	z		
**Dys > HC**	Vermis 7	2	−80	−24	35	3.2773
	Lingual L	−6	−82	−2	32	3.1681
	Calcarine R	12	−66	10	36	3.8273
**HC > Dys**	Cuneus R	12	−84	46	57	−3.5002
**EDys > HC**	Cerebelum 8 L	−36	−48	−48	146	3.8862
	Calcarine R	6	−88	4	79	4.1402
	Lingual R	24	−46	−2	60	4.9231
	Calcarine R	10	−74	12	319	3.7211
**HC > EDys**	Fusiform L	−26	−6	−50	62	−3.4123
	Temporal Pole Mid. R	8	8	−28	402	−3.8254
	Temporal Inf. L	−58	−40	−24	60	−3.1095
**Dys > EDys**	Fusiform L	−16	−2	−46	512	3.6009
	Fusiform R	24	−6	−48	241	3.7123
	Cerebelum 6 R	48	−40	−32	52	3.5915
	Temporal Inf. R	60	−50	−14	53	3.3171
**EDys > Dys**	Frontal Mid. Orb. R	34	36	−16	33	−3.5921
	Frontal Med. Orb. L	−8	62	−6	38	−3.2888
	Frontal Mid. L	−28	50	2	40	−3.4668
	Frontal Sup. R	18	64	8	38	−3.603
	Frontal Sup. L	−24	62	14	46	−3.4912
	Calcarine R	10	−68	18	48	−2.9935

* Inf: Inferior, * Orb: Orbital, *Med: Medial. The explanations written in Table 2 are valid.

**Table 4:** fALFF analysis of brain activity differences between groups.

Groups	Brain regions (AAL Atlas)	Peak MNI coordinates			Voxel size	Peak intensity
		x	y	z		
**Dys > HC**	Insula R	40	6	−12	39	3.7956
	Calcarine R	16	−74	6	80	3.7326
	Lingual R	24	−60	−2	36	3.0816
**HC > Dys**	Frontal Med. Orb. L	−2	56	−10	51	−3.9013
	Precuneus L	−4	−56	10	32	−3.5932
	Postcentral R	24	−34	54	30	−3.7709
**EDys > HC**	Temporal Mid. L	−48	−64	−4	36	3.7572
	Calcarine R	16	−74	12	99	3.8346
	Lingual R	26	−48	−4	47	3.5814
	Calcarine L	−14	−72	6	41	4.1019
	Calcarine L	−4	−76	8	40	2.9465
	Cingulum Mid. L	−2	6	42	32	3.4586
**HC > EDys**	Temporal Mid. R	50	−68	18	30	−4.0795
	Occipital Mid. L	−38	−78	36	32	−3.3464
**Dys > EDys**	Frontal Inf. Tri. L	−44	16	18	37	3.7088
**EDys > Dys**	Cerebelum 8 L	−42	−54	−56	31	−3.6881
	Frontal Med. Orb. L	−2	56	−10	47	−3.1897
	Precuneus L	−2	−74	38	32	−3.7074
	Cingulum Mid. L	−2	12	40	44	−3.2446

The explanations written in Table 2 and Table 3 are valid.

## Data Availability

This research was carried out under the TÜBİTAK project, and data sharing is currently restricted.
